# Enhancement of Perylenequinonoid Compounds Production from Strain of *Pseudoshiraia conidialis* by UV-Induced Mutagenesis

**DOI:** 10.3390/microorganisms13091999

**Published:** 2025-08-27

**Authors:** Xin Tong, Xiao-Ye Shen, Man-Rong Huang, Cheng-Lin Hou

**Affiliations:** 1 Department of Life Sciences, Natural History Museum of China, Tianqiaonandajie 126, Dongcheng, Beijing 100050, China; tongxin1112@126.com (X.T.); huangmanrong@nnhm.org.cn (M.-R.H.); 2 College of Life Science, Capital Normal University, Xisanhuanbeilu 105, Haidian, Beijing 100048, China; chenglin-hou@cnu.edu.cn

**Keywords:** perylenequinonoid compounds, UV mutagenesis, *Shiraia*-like fungi, fermentation period, HPLC

## Abstract

Perylenequinonoid compounds, represented by photosensitive therapeutic agents such as hypocrellins and elsinochromes, demonstrate extensive potential across biomedical, agricultural, and food industrial applications. Nevertheless, their restricted biosynthesis remains a critical bottleneck for commercial exploitation. This study implemented UV mutagenesis to enhance perylenequinone production in fungal strains of *Pseudoshiraia conidialis*, achieving significant yield improvements at the 120 J/m^2^ and 150 J/m^2^ irradiation intensities. Through systematic optimization of the HPLC analytical platform, we established the precise quantification of five distinct perylenequinonoid derivatives: hypocrellin A, hypocrellin B, shiraiachrome A, elsinochrome A, and elsinochrome B. The mutant strain Z2-1 demonstrated a remarkable biosynthetic capacity with the total perylenequinonoid yields reaching 2101.6 mg/L, representing a 705.70% enhancement over the parental strain zzz816 (260.84 mg/L). Particularly noteworthy was the hyperproduction of hypocrellin A at 1100.7 mg/L, corresponding to a 1208.02% increase from the baseline yield (84.15 mg/L). Furthermore, this work reports the first successful generation of an elsinochrome A-overproducing strain, achieving a 312.68 mg/L output (429.25% increase from 59.08 mg/L). Intriguingly, different mutant strains exhibited distinct production profiles for specific compounds, revealing biosynthetic preference variations among derivatives. These findings emphasize the necessity for comprehensive metabolite profiling during fermentation process optimization to maximize the target compound yields.

## 1. Introduction

**Figure 1:**
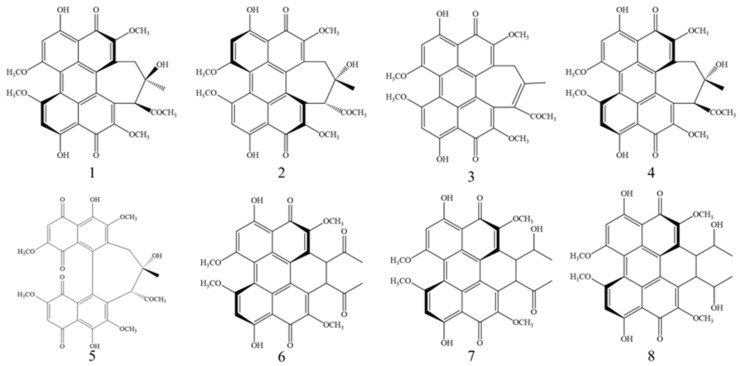
Chemical structures of hypocrellin (1), hypocrellin A (2), hypocrellin B (3), shiraiachrome A (4), hypocrellin D (5), elsinochrome A (6), elsinochrome B (7), and elsinochrome C (8).

Perylenequinonoid compounds belong to a class of pigments with photosensitive activity, covering hypocrellins, elsinochromes, phleichrome, cercosporin, and calphotins [1,2], and structures of these compounds are shown in Figure 1. Hypocrellins play an important role in traditional therapy and are well-known as the new generation of photosensitizers. Initially isolated from *Rubroshiraia bambusae* D.Q. Dai & K.D. Hyde (Synonymy *Hypocrella bambusae* (Berk. & Broome) Sacc.), they are now primarily extracted from species associated with *Shiraia* [3,4,5,6,7]. To date, five components—hypocrellin, hypocrellin A, hypocrellin B, shiraiachrome A, and hypocrellin D—have been identified in this group [8,9,10], with various bioactivities in the pharmaceutical, food, and agricultural industries [11,12,13]. Hypocrellin A (HA), the most prominent constituent, has demonstrated excellent light-induced antimicrobial, antiviral, and anticancer activities [14], with an especially significant efficacy against the HIV virus [15,16]. These activities are characteristic of photodynamic therapy (PDT) [17]. Recent studies have tackled HA’s poor water solubility through the use of poly(lactic-co-glycolic) acid nanoparticles or synthesizing targeted drug delivery systems (TDDS), significantly enhancing its PDT efficacy [18,19]. Although hypocrellin B (HB) shares a similar structure with HA [20], the presence of a hydroxyl group suggests a unique role. HB and its derivatives have been shown to induce tumor cell apoptosis and suppress cell viability, thereby contributing to their antitumor activity [21,22,23]. For instance, HB-LED PDT treatment significantly induced apoptosis in keloid fibroblasts (KFBs) and reduced KFB cell viability [24].

Elsinochromes, another class of perylenequinonoid compounds, are mainly isolated from *Elsinoe* [25,26] and can effectively combat pests and diseases without direct toxicity or side effects on the human body. There are also four components—elsinochrome A, B, C, and D—in this group [27], which have diverse effects on the corresponding field. Elsinochrome A (EA), which can now be artificially biosynthesized, is recognized as an excellent photosensitizer in the visible region, capable of effectively killing microorganisms, cells, and viruses [28]. Moreover, EA’s quantum yield of singlet oxygen surpasses that of other photosensitizers [29]. Elsinochrome C (EC) also exhibits phytotoxicity through light induction [30].

Despite their significant potential, the limited production of perylenequinonoid compounds still poses a major challenge, hindering further advancements in their research and application. To overcome the bottleneck, high-yield strains have been screened from diverse plant resources [31,32,33], and submerged fermentation conditions have been optimized from different aspects [34,35]. Recent studies have employed mutagenesis breeding to enhance the production of perylenequinonoid compounds including cobalt mutagenesis [36], nitrosoguanidine mutagenesis [37], and calcium/calmodulin signal transduction mutagenesis [38]. Moreover, some significant advances have been achieved in the biosynthetic pathway of hypocrellins, particularly in deciphering the enzymatic function of polyketide synthase and the transcriptional regulatory network via key transcription factors [33,39]. However, these efforts have yet to meet the industrial demands for large-scale production.

Notably, according to the latest taxonomic studies, all of the hypocrellin-yielding strains should be classified into *Shiraia*-like fungi (*Pseudoshiraia conidialis*) [32]. Mitogenomic analyses further revealed that these industrial strains comprised two major groups, zzz816 and JAP103846, which separately exhibit diverged regulatory mechanisms of hypocrellin biosynthesis [40]. Based on this, we found that the vast majority of engineered strains covering mutagenesis breeding, molecular transformation, and cultivation optimization could be predominantly attributed to the JAP103846 group, while the strains associated with zzz816 have attained little attention. In other words, the special transcription factor *SbTF*, located on the gene cluster for hypocrellin synthesis, also indicates distinct transcriptional regulation between zzz816 and JAP103846 [33]. In particular, the zzz816 group possesses a constitutive gene expression and stably produces natural products unlike JAP103846, which still depends on inducible elicitor-induced activation [32,35,36,41]. Therefore, the zzz816 group demonstrates greater potential for further enhancement in genetic breeding and industrial application.

Although ultraviolet (UV) mutagenesis has been widely employed as a conventional technique in fungal strain improvement, this approach still remains deficient in zzz816 strains. Emerging evidence has revealed that UV light can directly trigger oxidative stress responses and reactive oxygen species (ROS) accumulation within the fungal hyphae, while hypocrellin biosynthesis demonstrates a positive correlation with intracellular ROS levels and can be upregulated by oxidative inducers such as H_2_O_2_ or NO [39,42,43,44,45,46]. Therefore, it is anticipated that UV mutagenesis would not only introduce genomic modifications, but also simultaneously establish a favorable condition for improving hypocrellin production. Interestingly, while certain groups of perylenequinonoid compounds are typically produced by a limited number of species (e.g., cercosporins from *Cercospora* [47]), *Shiraia*-like fungi exhibit a unique capability to synthesize diverse groups of these compounds [48,49]. In some subgroups, their crude extracts contain not only hypocrellins, but also a certain amount of elsinochromes. Hypocrellin A, hypocrellin B, shiraiachrome A, and elsinochrome A, B, C, and D each possess distinct structures and exhibit diverse bioactivities. When multiple agents coexist in the final product, focusing solely on hypocrellin A or the total hypocrellins is insufficient.

In this study, we utilized UV radiation to enhance the yield of perylenequinonoid compounds, and through optimized high performance liquid chromatography (HPLC) analysis, we simultaneously screened the production of different perylenequinonoid components.

## 2. Materials and Methods

### 2.1. Strains and Culture Conditions

The original strain *Pseudoshiraia conidialis* zzz816 (ACCC38984) was isolated from moso bamboo seeds [31] and cultured on 2% potato dextrose agar medium (PDA, containing 200 g/L potato, 20 g/L dextrose, and 20 g/L agar; pH 6.0) at 25 °C in the dark. The fungal strain was stocked in the Agricultural Culture Collection of China (ACCC).

Fresh mycelia of fungal strain zzz816 were cultured on PDA plates for 7 days at 25 °C. Six plugs (6 mm in diameter) of the growing culture, along with adhering mycelia, were transferred to 250-mL Erlenmeyer flasks containing 100 mL potato dextrose broth medium (PDB, containing 200 g/L potato and 20 g/L dextrose; pH 6.0). Liquid cultures were maintained at 26 °C for 60 h with shaking (150 rpm). A 0.1 mL aliquot of the fermentation broth was evenly spread onto PDA plates and incubated at 25 °C for 7 days [36].

### 2.2. Preparation of Spore Suspension and UV Mutagenesis

The PDA plates were separately submerged in 10 mL of sterile ultra-pure water (containing 0.02% Tween-80), and the spores were suspended to a concentration of 10^6^ spores/mL [50]. The spore suspensions were then exposed to varying doses of UV radiation (100 J/m^2^, 120 J/m^2^, 150 J/m^2^, 300 J/m^2^, 800 J/m^2^, and 1000 J/m^2^) at room temperature. The distance between the spore suspensions and the UV lamp was 30–40 cm, and these were exposed for 60 s, 90 s, 100 s, 3 min, 8 min, and 10 min separately. After exposure, the spore suspension was uniformly spread onto PDA medium and incubated at 25 °C for 7 days in the dark [36,37,38]. Three independent biological replicates were performed for each treatment.

### 2.3. Lethality Assay

Spore mortality was analyzed to determine the lethality of different UV intensities. Survival data were fitted to the below equation

S = D_0_/D × 100%,

and the lethality data to the equation

L = (D − D_0_)/D × 100%,

where S, L, D, and D_0_ represent the survival fraction, lethality fraction, total number of viable colonies before UV treatment, and number of viable colonies after UV treatment, respectively.

### 2.4. Preliminary Screening of High-Yield Mutants

Following the germination of UV-treated spores, five mutant strains were randomly selected from each PDA plate exposed to different UV intensities. These strains were then inoculated onto new PDA plates and incubated at 25 °C for 7 days in the dark for preliminary screening.

Perylenequinonoid compounds are shown as red pigments under visible light, enabling direct morphological observation for screening high-yield strains. Next, five strains were individually selected for further study based on the growth rate and pigment.

### 2.5. Growth Rate Determination and Microscopic Observations of Mycelial Morphology

The original strain zzz816 and the mutant strains were simultaneously inoculated onto PDA plates and cultured at 25 °C in the dark for 7 days. The colony diameters of each strain were measured and the mycelial morphology of each strain was observed using a light microscope (Olympus DP71, Tokyo, Japan).

### 2.6. Analysis of Fermentation Time and Mycelial Biomass

The original strain zzz816 and the mutant strains were inoculated into 250-mL Erlenmeyer flasks containing 150 mL PDB liquid medium and shaken at 180 rpm at 26 °C. Mycelia were collected at different time points (5–10 days), filtered, freeze-dried, and weighed to plot the growth curves.

### 2.7. Extraction of Intracellular Perylenequinonoid Compounds

The original strain zzz816 and the selected mutant strains were inoculated onto PDA plates and incubated for 3 days, then transferred to PDB medium as described above. After 7 days of cultivation, the fermented mycelia were harvested by centrifugation at 12,000 rpm for 10 min at 4 °C, rinsed three times with distilled water, and vacuum freeze-dried. The mycelia were ground into powder using liquid nitrogen, and 0.5 g of the mycelium pellets was accurately weighed and chemically extracted with 150 mL of absolute ethanol via Soxhlet extraction for 12 h at 95 °C. The ethanol was evaporated under vacuum at 45 °C, and the residues were dissolved in 10 mL methanol for HPLC analysis.

### 2.8. HPLC Analysis Conditions

The content of perylenequinonoid compounds in the extract was analyzed using an Agilent 1200 Series high performance liquid chromatography system equipped with a Kromasil 100-5C18 (250 × 4.6 mm) column (Nouryon, Bohus, Sweden). The operating conditions included the flow rate of 1.0 mL/min, the column temperature of 35 °C, and the sample volume of 20 μL. The detection wavelength was 460 nm. The elution gradient is detailed in Table 1 [32].

### 2.9. Analysis of Perylenequinonoid Compounds

Standards of hypocrellin A (HA), hypocrellin B (HB), and shiraiachrome A (SA), purity ≥ 98% (HPLC), were purchased from Biopurify Phytochemicals Ltd. (Chengdu, China). Standards of elsinochrome A (EA), elsinochrome B (EB), purity ≥ 98% (HPLC), were purchased from Hangzhou Viablife Biotech Co. Ltd. (Hangzhou, China). The standard samples of perylenequinonoid compounds were analyzed by HPLC. Their identification was confirmed by comparing the retention times and spectroscopic data [32]. Quantitative analysis was performed using standard curves, prepared according to the method described by Tong et al. [48]. More detailed information about the methodology of quantification is provided in the Supplementary Materials.

### 2.10. Genetic Stability Test of Perylenequinonoid Compounds from Mutant Strains

To verify the stability of perylenequinonoid compound production, mutant strains were cultured continuously on PDB medium for five generations. The content of perylenequinonoid compounds was analyzed by HPLC for each generation.

## 3. Results

### 3.1. Selection of Mutant Strains and Lethality Analysis

**Figure 2:**
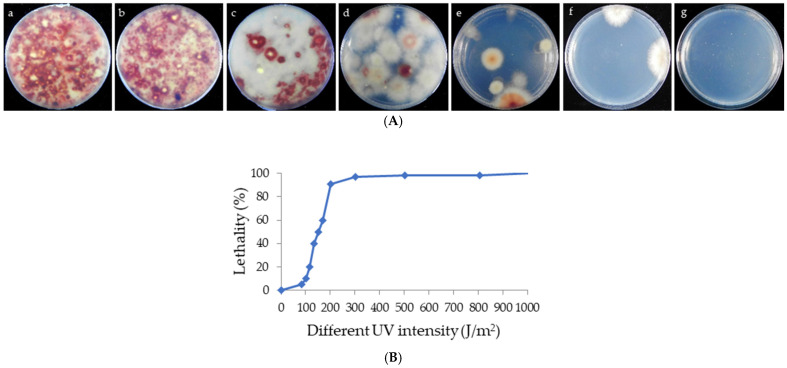
(**A**) Germination of spores under different UV intensities. (**a**) Germination of original strain zzz816. (**b**–**g**) Germination of spores treated with UV intensities of 100 J/m^2^, 120 J/m^2^, 150 J/m^2^, 300 J/m^2^, 800 J/m^2^, and 1000 J/m^2^, respectively. (**B**) Lethality of spores treated with different UV intensities.

Spores of *Pseudoshiraia conidialis* were treated with various UV intensities (100 J/m^2^, 120 J/m^2^, 150 J/m^2^, 300 J/m^2^, 800 J/m^2^, and 1000 J/m^2^). Lethality rates were calculated based on the number of colonies surviving after UV exposure, incubated at 25 °C for 3 days on PDA medium (Figure 2A). Figure 2B shows that spore lethality increased with the UV intensity. When the spores were treated with UV intensity from 100 J/m^2^ to 300 J/m^2^, the lethality of spores was enhanced sharply; at 150 J/m^2^, over half of the spores were non-viable, and no spores survived at 1000 J/m^2^.

### 3.2. Preliminary Screening of High-Yield Mutant Strains

As listed in Table 2, the strains were treated with different UV intensities. Then, based on a comprehensive evaluation including the growth rate and mycelial pigment, Z1-2, Z2-1, Z3-1, and Z4-1 were selected for further study.

### 3.3. Determination of Growth Rate

**Figure 3:**
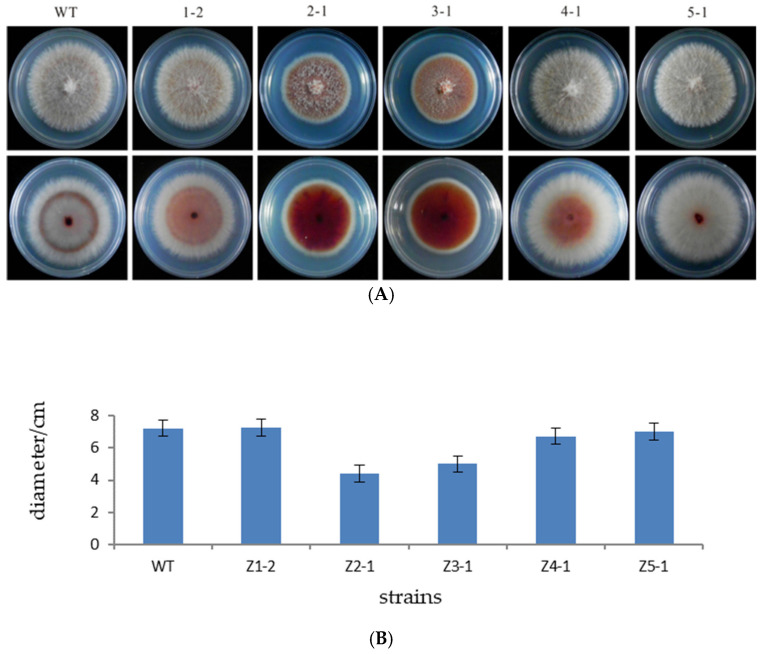
Growth rate determination of the strains. (**A**) Growth phenotypes of the wild-type and mutant strains. The top row shows front views of 7-day-old PDA cultures of the original strain zzz816 (WT) and the mutant strains (Z1-2, Z2-1, Z3-1, Z4-1, and Z5-1). The bottom row displays the corresponding back views of the cultures. (**B**) Growth rate of the original strain zzz816 and the mutant strains.

The original strain zzz816 and the mutant strains (Z1-2, Z2-1, Z3-1, Z4-1, and Z5-1) were simultaneously inoculated onto PDA plates and cultured at 25 °C in the dark for 7 days. Colony diameters (Figure 3A) were measured to analyze the growth rates. The growth rates of the mutant strains Z2-1 and Z3-1 were significantly reduced by 39.14% and 30.84%, respectively (Figure 3B).

### 3.4. Microscopic Observations of Mycelium Morphology

**Figure 4:**
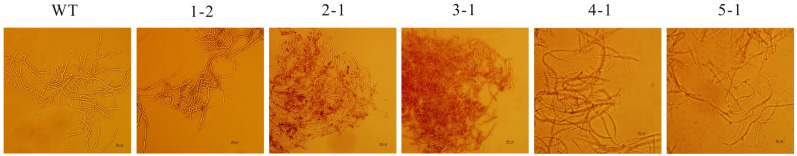
Mycelia of the original strain zzz816 (WT) and the mutant strains (Z1-2, Z2-1, Z3-1, Z4-1, and Z5-1) treated with different UV intensities under the microscope.

Microscopic examination revealed that the original strain’s mycelia were more filamentous, while the mycelia of the Z2-1 and Z3-1 mutants were notably spherical, thicker, and shorter (Figure 4).

### 3.5. Analysis of Fermentation Time and Mycelia Biomass

**Figure 5:**
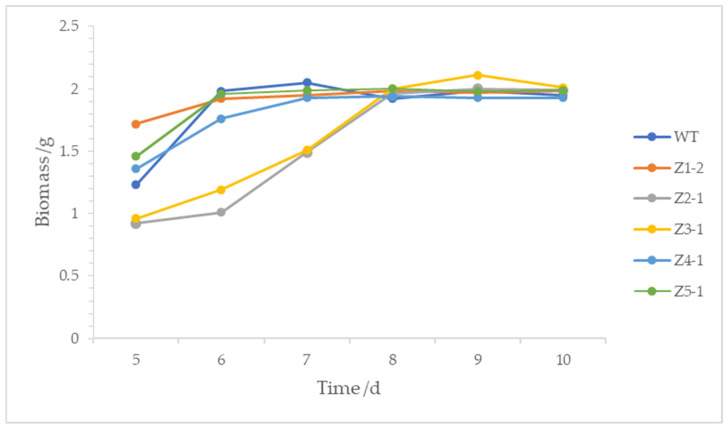
Fermentation periods of the original strain zzz816 (WT) and the mutant strains (Z1-2, Z2-1, Z3-1, Z4-1, and Z5-1).

As shown in Figure 5, compared with the original strain zzz816, Z1-2 exhibited a longer fermentation period, with maximum biomass on the eighth day. Z2-1 and Z3-1 also had extended fermentation periods, with the biomass increasing rapidly from the fifth to the eighth day before stabilizing. Z5-1 showed a similar pattern, reaching maximum biomass on the sixth day. Z4-1’s fermentation period remained unchanged, with maximum biomass on the seventh day. Overall, the maximum biomasses of the mutant strains did not significantly differ from the original strain.

### 3.6. Identification and Analysis of Perylenequinonoid Compounds

**Figure 6:**
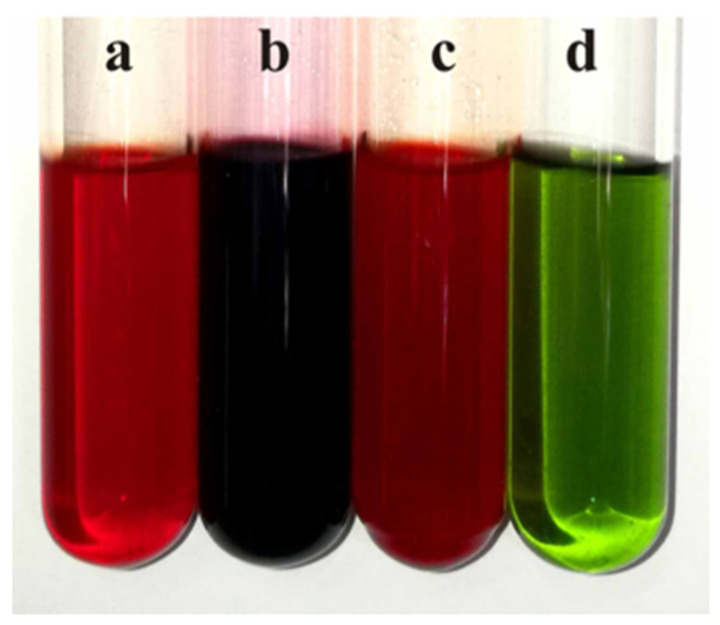
Chemical color response test of the extracts. (**a**) Ethanol extract of the original strain zzz816. (**b**) Extract with FeCl_3_ solution. (**c**) Extract with acidic solution. (**d**) Extract with alkaline solution.

Chemical color response tests were employed to verify the perylenequinonoid compounds in mutant strains. Extracts containing such compounds turned black with FeCl_3_ solution, red with acidic solutions, and green with alkaline solutions (Figure 6).

**Figure 7:**
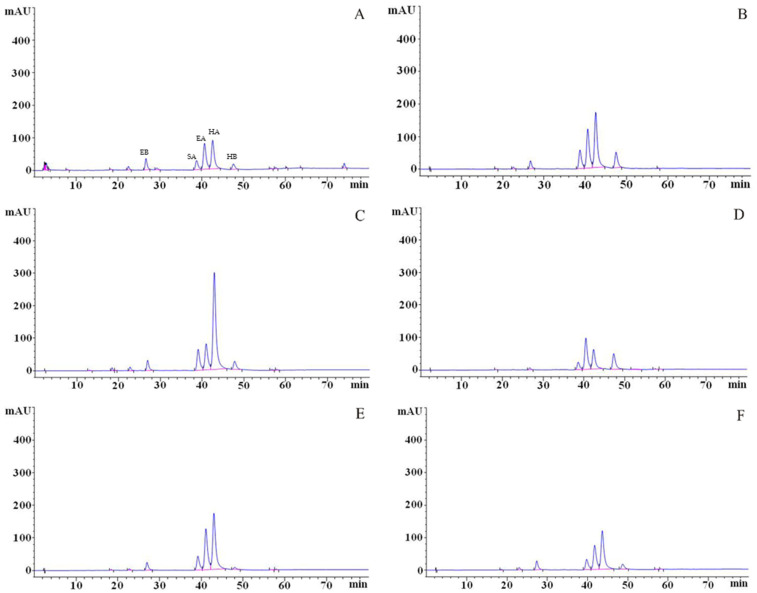
HPLC chromatograms of the samples. (**A**) Original strain zzz816. (**B**) Mutant strain Z1-2. (**C**) The sample of mutant strain Z2-1 after fourfold dilution. (**D**) The sample of mutant strain Z3-1 after fourfold dilution. (**E**) Mutant strain Z4-1. (**F**) Mutant strain Z5-1.

The perylenequinonoid compounds were identified through HPLC analysis (Figure 7), with characterization based on a comparative evaluation of UV–Vis spectra, chromatographic profiles, and retention times against standards [32]. Five sharp peaks were observed in the HPLC chromatogram of the original strain zzz816: elsinochrome B (EB) at 26.331 min, shiraiachrome A (SA) at 38.129 min, elsinochrome A (EA) at 39.955 min, hypocrellin A (HA) at 41.912 min, and hypocrellin B (HB) at 46.808 min.

### 3.7. Quantitative Determination of Perylenequinonoid Compounds

**Figure 8:**
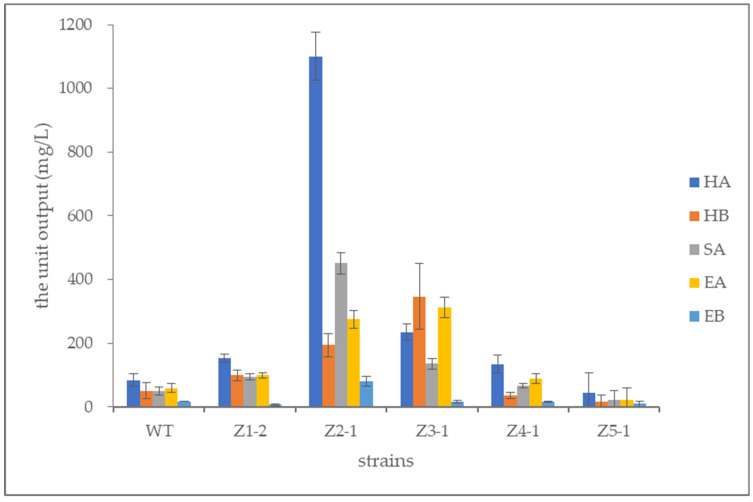
Perylenequinonoid compounds in the original strain and mutant strains.

Standard curves were plotted using perylenequinonoid compound standards. For each sample, the content of each perylenequinonoid compound and the total content were calculated (Table 3 and Table 4, and Figure 8). Results showed that Z2-1 and Z3-1 produced higher amounts of perylenequinonoid compounds, corresponding to the UV treatments with intensities of 120 J/m^2^ and 150 J/m^2^, respectively.

Among the mutants, Z2-1 exhibited the highest total yield of perylenequinonoid compounds at 2101.6 mg/L, a 705.70% increase over the original strain (261.20 mg/L). Hypocrellin A production in Z2-1 reached 1100.7 mg/L, a 1208.02% increase from the original strain (84.15 mg/L). The hypocrellin B yield was 194.02 mg/L, a 286.88% increase from the original strain (50.15 mg/L). The shiraiachrome A yield was 451.20 mg/L, an 806.21% increase from the original strain (49.79 mg/L), while the elsinochrome A yield was 275.28 mg/L, a 365.95% increase from the original strain (59.08 mg/L). The elsinochrome B yield was 80.40 mg/L, a 355.01% increase from the original strain (17.67 mg/L) (Figure 8).

Z3-1 produced 312.68 mg/L of elsinochrome A, a 429.25% increase from the original strain (59.08 mg/L). Notably, elsinochrome A production in Z3-1 exceeded that of other components. Additionally, hypocrellin B production in Z3-1 significantly increased, surpassing hypocrellin A production—a phenomenon not previously reported. Through the analysis, we found not only that hypocrellin A significantly increased, but also that other types of perylenequinonoid compounds had also been greatly improved and displayed a high yield. Furthermore, the production of hypocrellin A and the total perylenequinonoid compounds in Z2-1 reached high levels (Figure 8).

### 3.8. Genetic Stability of Perylenequinone-Producing Mutant Strains Z2-1 and Z3-1

The high-yielding perylenequinone-producing mutant strains, designated as Z2-1 and Z3-1, were subjected to a stability evaluation through five successive subcultures. The production stability of perylenequinonoid compounds in these mutants was subsequently analyzed using HPLC. Results demonstrated that despite occasional minor fluctuations, both mutant strains maintained steady production yields through five generations, as detailed in Table 5.

## 4. Discussion

UV mutagenesis, known for its reliability, simplicity, and significant mutagenic results, has been widely used in microbial breeding. For example, it has been employed to enhance vitamin B12 production in *Propionibacterium freudenreichii* (*Propionibacterium shermanii*) [51] and nucleoside production in *Cordyceps kyushuensis* [52]. A previous study conducted complex mutagenesis and increased hypocrellin A production in *Shiraia bambusicola* to 80.4 mg/L, a 167.1% increase over the original strain [37]. Cobalt mutagenesis in *Shiraia* sp. achieved a hypocrellin A production of 2018.30 mg/L [36], and calcium/calmodulin signal transduction improved the perylenequinonoid compound yield to 1894.66 mg/L [38].

However, a significant gap still exists in strain breeding around the zzz816 group. Furthermore, a recent report revealed the distinct transcriptional regulation mechanisms between zzz816 and JAP103846 [33,39]. This suggests that zzz816-related strains would possess greater potential for genetic modification. Combined with their inherent advantages, including a higher native hypocrellin yield and the absence of inducer requirements [31,36,40], these strains indicate considerable potential for production applications.

In this study, UV irradiation was employed for the mutagenesis strategy due to its dual role in inducing DNA mutation and triggering the oxidative stress response. Crucially, it has been proven that the appearance of ROS could enhance hypocrellin biosynthesis in *Shiraia*-like fungi [43,44,45,46]. As anticipated, this single-factor UV mutagenesis yielded successfully multiple mutants with improved hypocrellin production. This outperformed many JAP103846 strains with complex, multi-factor mutagenesis approaches, highlighting the greater efficiency of this method in *Shiraia*-like fungi.

Furthermore, some promising mutants, covering Z2-1 and Z3-1, exhibited a growth pattern similar to that observed in JAP103846: enhanced intracellular hypocrellin production correlates with increased growth inhibition. This inhibitory effect may be attributed to the intrinsic photosensitizing properties of hypocrellins, which can interfere directly with normal fungal growth [53,54]. However, it is encouraging that the growth rates of the Z2-1 and Z3-1 mutants were not hindered as badly as the engineered JAP103846, and the screening of faster-growing variants during serial subculturing can effectively compensate for the deficiency.

It is noteworthy that using only a straightforward UV-mutagenesis approach, we collected several mutants with significantly enhanced performance and commercial value. This demonstrates that isolates from the zzz816 clade may more effectively utilize a metabolic regulatory mechanism distinct from the other strains. Correspondingly, the special transcription factor *SbTF* for hypocrellin synthesis also performs different regulatory functions across various genetic backgrounds [33], while mitochondrial genome-encoded nad and cox family proteins show distinct expression patterns [40]. Likewise, the analysis of transcriptome data from different sources highlighted substantial differences in the core genes for hypocrellin synthesis (NCBI Sequence Read Archive (SRA) database with the BioProject accession numbers PRJNA323638, PRJNA475310, PRJNA477419, and PRJNA544773). Moving forward, we plan to reorganize published omics data according to current molecular classifications and combine them with the expression changes of key genes from high-yield strains to better understand the underlying regulatory mechanism.

However, these studies focused solely on hypocrellin A or crudely estimated mixed perylenequinonoid compounds. Our results demonstrate that other perylenequinonoid compounds also play significant roles in the final products, sometimes even surpassing hypocrellin A in certain strains like Z2-1 and Z3-1. Neglecting these compounds could lead to significant waste and inconsistent product quality. In this study, a modified analytical system was used to simultaneously quantify five different perylenequinonoid compounds (HA, HB, SA, EA, and EB). The results revealed diverse proportions of these compounds in different mutants, with elsinochrome A surpassing hypocrellin A in Z3-1. Therefore, focusing solely on hypocrellin A underestimates the productive capacity of these strains. Given the distinct properties and effects of these compounds, particularly between hypocrellins and elsinochromes, it is crucial to measure each component separately. This study’s approach, simultaneously detecting each component, lays a solid foundation for evaluating diverse strains with unique properties.

In this study, all mutants achieved similar maximum biomass levels under the same fermentation conditions. From a theoretical perspective, shorter fermentation times are more beneficial. To optimize time and energy, future research should focus on improving the media composition and submerged fermentation conditions through statistically designed experiments.

UV irradiation is a conventional mutagenesis approach that induces mutations to enhance production while maintaining normal growth pattern. These strains can generally still undergo further optimization through transgenic modifications, fermentation adjustments, and EMS [33,39,49]. The UV-mutagenized variants also exhibit substantial potential for further improvement via compound mutagenesis.

## Figures and Tables

**Table 1:** The 0.1% phosphoric acid water–methanol phase of the HPLC gradient.

Time (min)	0.1% Phosphoric Acid Water (%, *v*/*v*)	Methanol (%, *v*/*v*)
0–10	40	60
10–15	40–30	60–70
15–25	30	70
25–45	30–25	70–75
45–60	25–0	75–100
60–80	0	100

**Table 2:** Strains treated with different UV intensities.

UV Intensity (J/m^2^)	Strain Names
100	Z1-1, Z1-2, Z1-3, Z1-4, Z1-5
120	Z2-1, Z2-2, Z2-3, Z2-4, Z2-5
150	Z3-1, Z3-2, Z3-3, Z3-4, Z3-5
300	Z4-1, Z4-2, Z4-3, Z4-4, Z4-5
800	Z5-1, Z5-2, Z5-3, Z5-4, Z5-5

**Table 3:** Perylenequinonoid compounds (mg/g) in the original and mutant strains during 7-day cultivation.

Strains	HA	HB	SA	EA	EB	Total Content
WT	6.16 ± 1.38	3.67 ± 1.83	3.64 ± 0.82	4.32 ± 0.44	1.29 ± 0.06	19.09 ± 4.54
Z1-2	11.78 ± 1.01	7.64 ± 1.24	7.26 ± 0.70	7.66 ± 0.63	0.57 ± 0.25	34.91 ± 3.81
Z2-1	110.81 ± 7.69	19.53 ± 3.65	45.42 ± 3.43	27.71 ± 2.77	8.09 ± 1.56	211.57 ± 19.10
Z3-1	23.35 ± 2.47	34.44 ± 10.25	13.41 ± 1.56	31.06 ± 3.10	1.60 ± 0.31	103.87 ± 17.69
Z4-1	10.49 ± 2.10	2.70 ± 0.78	5.17 ± 0.58	6.89 ± 1.23	1.26 ± 0.13	26.50 ± 4.81
Z5-1	3.23 ± 4.79	1.30 ± 1.49	1.63 ± 2.30	1.72 ± 2.79	0.71 ± 0.64	8.59 ± 12.01

HA: hypocrellin A, HB: hypocrellin B, SA: shiraiachrome A, EA: elsinochrome A, EB: elsinochrome B.

**Table 4:** Perylenequinonoid compounds (mg/L) in the original and mutant strains during 7-day cultivation.

Strains	HA	HB	SA	EA	EB	Total Content
WT	84.15 ± 18.89	50.15 ± 25.06	49.79 ± 11.20	59.08 ± 14.34	17.67 ± 0.79	260.84 ± 70.28
Z1-2	153.18 ± 13.10	99.29 ± 16.12	94.34 ± 9.04	99.61 ± 8.14	7.39 ± 3.20	453.81 ± 49.59
Z2-1	1100.70 ± 76.40	194.02 ± 36.21	451.20 ± 34.04	275.28 ± 27.50	80.40 ± 15.49	2101.60 ± 189.64
Z3-1	235.08 ± 24.83	346.68 ± 103.25	135.04 ± 15.72	312.68 ± 31.23	16.12 ± 3.11	1045.60 ± 178.14
Z4-1	134.91 ± 27.02	34.75 ± 10.06	66.46 ± 7.43	88.59 ± 15.82	16.23 ± 1.62	340.94 ± 61.95
Z5-1	42.91 ± 63.56	17.20 ± 19.79	21.63 ± 30.50	22.87 ± 37.09	9.38 ± 8.51	113.99 ±159.43

HA: hypocrellin A, HB: hypocrellin B, SA: shiraiachrome A, EA: elsinochrome A, EB: elsinochrome B.

**Table 5:** Yield of the total perylenequinonoid compounds in mutant strains Z2-1 and Z3-1 over five generations.

Generation Numbers	Mutant Strain Z2-1 (mg/L)	Rate of Change (%)	Mutant Strain Z3-1 (mg/L)	Rate of Change (%)
1	2101.6	0	1045.6	0
2	2198.7	4.62	1094.3	4.66
3	2237.5	6.47	1147.8	9.77
4	2045	−2.69	978.2	−6.45
5	2219.2	5.60	1121.8	7.29

## Data Availability

The original contributions presented in this study are included in the article/Supplementary Materials. Further inquiries can be directed to the corresponding author.

## Supplementary Materials

The following supporting information can be downloaded at: https://www.mdpi.com/article/10.3390/microorganisms13091999/s1, Table S1: Concentrations of the standard stock solutions, Table S2: Results of the linear regression analysis, Table S3: Precision evaluation of HPLC method for perylenequinone standard analysis, Table S4: Stability evaluation of perylenequinone standards over 24 h.
