# Supplementary material for: Enhancement of Perylenequinonoid Compounds Production from Strain of Pseudoshiraia conidialis by UV-Induced Mutagenesis
Source: Microorganisms. 2025 Aug 27;13(9):1999. doi: 10.3390/microorganisms13091999 (PMC12471705; doi:10.3390/microorganisms13091999)
Supplement: Supplementary file 1 [file microorganisms-13-01999-s001.zip › microorganisms-3745301-supplementary.pdf]

Supplementary Materials

S1 HPLC Methodological Investigation for the Quantification of Perylenequinonoid Compounds

S1.1 Linearity

Standard stock solutions of hypocrellin A (HA), hypocrellin B (HB), shiraiachrome A (SA), elsinochrome A (EA), and elsinochrome C (EC) were prepared by dissolving accurately weighed standards in methanol and serially diluting into the following concentrations (Table S1).

Table S1. Concentrations of the standard stock solutions

Standard	Weight (mg)	Concentrations (mg/ml)
HA	1.12	0.0448, 0.112, 0.224, 0.336, 0.448, 0.56
HB	0.72	0.018, 0.036, 0.072, 0.108, 0.144, 0.18, 0.216
SA	0.84	0.0168, 0.042, 0.084, 0.126, 0.168, 0.21, 0.252
EA	1.80	0.045, 0.09, 0.18, 0.27, 0.36, 0.45, 0.54
EB	1.00	0.025, 0.05, 0.1, 0.15, 0.2, 0.25, 0.3

Linear regression analysis of peak area (y) versus concentration (x) yielded the calibration equations with high correlation coefficients (R^2) (Table S2). The results demonstrated the excellent linearity for all analytes.

Table S2. Results of the linear regression analysis

Standard	Calibration Equation	R^2
HA	y = 20253 x - 34.23	1
HB	y = 8100.6 x - 5.8125	0.9995
SA	y = 9508.9 x + 23.105	0.9998
EA	y = 8594.4 x - 36.844	0.9997
EB	y = 18844 x - 4.0542	1

S1.2 Precision

Method precision was assessed by injecting each standard solution (10 μL) five consecutive times. The relative standard deviations (RSD) of the peak areas, as summarized in Table S3, were all ≤1.26%, demonstrating high repeatability of the method.

Table S3. Precision evaluation of HPLC method for perylenequinone standards analysis

Standard	Peak Area					Mean	RSD%
	1	2	3	4	5		
HA	11277.2	11286.7	11314.1	11336.7	11286.7	11300.28	0.19
HB	1716.5	1727.6	1727.1	1767	1728.7	1733.38	1.00
SA	2001.8	2016.8	2018.9	2025.1	2026	2017.72	0.43
EA	2352.8	2352.7	2333.5	2352.7	2353.5	2349.04	0.33
EB	461.5	462	461.6	463.1	447.7	459.18	1.26

S1.3 Stability

Analyte stability was assessed by injecting standard solutions at 0, 4, 8, 12, and 24 h. RSD values ranged from 0.78% to 1.55%, indicating stable analyte responses within 24 h (Table S4).

Table S4. Stability evaluation of perylenequinone standards over 24 h

Standard	Peak Area					Mean	RSD%
	0 h	4 h	8 h	12 h	24 h		
HA	2236.5	2192.3	2237.8	2264.1	2218.2	2229.78	1.07
HB	276.9	279.7	280.6	283.8	285.1	281.22	1.04
SA	1586	1628.1	1623.9	1590.4	1628	1611.28	1.18
EA	1441.3	1445.2	1473.3	1446.7	1449.2	1451.14	0.78
EB	938.2	913.9	948.2	919.7	948.7	933.74	1.55
